# Comma Bacillus

**Published:** 1885-05

**Authors:** 


### COMMA BACILLUS.

We have received, from Berlin, slides of the Cholera Comma Bacillus of Koch, and the Comma Bacillus of Miller, found in the human mouth, and of which he gives a description in this number. We will lend them to any of our readers who are bacteriologists, and who desire them for study, and not out of mere curiosity. Their proper examination will require first-class apparatus and manipulation.

